# Unresolved Trauma and Reorganization in Mothers: Attachment and Neuroscience Perspectives

**DOI:** 10.3389/fpsyg.2019.00110

**Published:** 2019-01-30

**Authors:** Udita Iyengar, Purva Rajhans, Peter Fonagy, Lane Strathearn, Sohye Kim

**Affiliations:** ^1^Institute of Psychiatry, Psychology, and Neuroscience, King’s College London, London, United Kingdom; ^2^Department of Pediatrics, Baylor College of Medicine, Houston, TX, United States; ^3^Stead Family Department of Pediatrics, University of Iowa Carver College of Medicine, Iowa City, IA, United States; ^4^Menninger Department of Psychiatry and Behavioral Sciences, Baylor College of Medicine, Houston, TX, United States; ^5^Anna Freud National Centre for Children and Families, London, United Kingdom; ^6^Research Department of Clinical, Educational, and Health Psychology, University College London, London, United Kingdom; ^7^Center for Disabilities and Development, University of Iowa Stead Family Children’s Hospital, Iowa City, IA, United States; ^8^Department of Obstetrics and Gynecology, Baylor College of Medicine, Houston, TX, United States; ^9^Center for Reproductive Psychiatry, Pavilion for Women, Texas Children’s Hospital, Houston, TX, United States

**Keywords:** attachment, reorganization, unresolved trauma, intergenerational transmission, maternal brain, neuroscience

## Abstract

The onset of motherhood is characterized by significant psychological and neurobiological changes. These changes equip the mother to care for her new child. Although rewarding, motherhood is also an inherently stressful period, more so for mothers with unresolved trauma. Past research has looked at how unresolved trauma can hamper a mother’s caregiving response toward her infant, which further affects the development of secure attachment in her own infant. The Dynamic Maturational Model of Attachment and Adaptation (DMM) has introduced a unique concept of “attachment reorganization” which can be described as a process whereby individuals with unresolved trauma are transitioning toward attachment security based on their enhanced understanding of past and present experiences. Preliminary results from one of our previous studies have shown that, among mothers with unresolved trauma, mothers who themselves demonstrated “reorganizing attachment” toward security, had infants with secure attachment, thereby indicating the potential to halt the intergenerational transmission of insecure attachment. While this concept is of great clinical relevance, further research is required to assess the benefits of attachment reorganization as a protective factor and its positive implications for child development. Thus, the aim of the current review is to expand on the concept of attachment reorganization in mothers with unresolved trauma from both attachment and neuroscience perspectives. To that effect, we will first review the literature on the transition to motherhood from attachment and neuroscience perspectives. Second, we will use attachment and neuroscience approaches to address deviations from normative experiences during motherhood with a specific focus on the role of a mother’s unresolved trauma. Lastly, we will expand on the concept of reorganization and the promise this concept holds in resolving or halting the intergenerational transmission of trauma from mothers to their children.

## Introduction

Mothers undergo significant adaptation and reorganization throughout pregnancy and postpartum, both on psychological and neurobiological levels. Psychologically, a new mother’s emotional investment is drawn away from the outside world and refocused inward toward her infant (Slade et al., 2009). Attachment theory is a useful framework to understand how these formative relationships shape our psychological organization and behavior across the lifespan. Specifically in the maternal caregiving context, attachment theory draws attention to the importance of a mother’s sensitive response to her infant as an antecedent to the child’s development of secure attachment in adulthood. Neurobiologically, a new mother undergoes neural and endocrine changes that prepare her to respond to her new infant’s all-encompassing needs (Swain et al., 2007; Kim et al., 2016; Swain and Ho, 2017).

Despite being an intrinsically rewarding experience for many mothers (Strathearn et al., 2008), motherhood comes with its set of challenges as the new mother adapts to the aforementioned changes. Moreover, these challenges can become heightened in the presence of early-life adversity, unresolved trauma, or psychopathology. These factors may alter a mother’s behavioral and neural response to her infant, interfering with the dyadic relationship and setting the stage for disrupted attachment across generations. Identifying potential protective factors may help mitigate this disrupted relationship between at-risk mothers and their infants, potentially halting the intergenerational transmission of insecure attachment.

“Attachment reorganization” is a unique concept introduced by the Dynamic Maturational Model of Attachment and Adaptation (DMM) method of coding the Adult Attachment Interview (AAI). It is defined as a process whereby individuals with unresolved trauma are transitioning toward attachment security based on their increased understanding and resolution of past and present traumatic experiences. We have previously reported that, among mothers with unresolved trauma, those who were “reorganizing” toward secure attachment were the only individuals with unresolved trauma whose children were determined to be securely attached, underscoring the potential of attachment reorganization in halting the intergenerational transmission of insecure attachment (Iyengar et al., 2014). The aim of the present paper is to further expand on our previous work (Iyengar et al., 2014) and evaluate the benefits of attachment reorganization, considering the literature from both attachment and neuroscience perspectives. In what follows, we will first explain adaptive transition to motherhood from attachment and neuroscience perspectives. Second, we will address circumstances that may interfere with adaptive mothering, such as the presence of unresolved trauma and associated clinical features or conditions, drawing from both attachment and neuroscience perspectives. Finally, we will explore protective factors that may halt the transmission of disrupted attachment across generations. For this purpose, we will specifically focus on the concept of attachment reorganization and its potential role in resolving or halting the intergenerational transmission of trauma from mothers to their children.

## Adaptive Pregnancy and Transition to Motherhood

### Attachment Perspectives

Maternal sensitivity requires a highly attuned level of reciprocity and synchrony, which is built over time and requires constant adaptation and reorganization (Ainsworth et al., 1978). D.W. Winnicott first saw the interactions between the mother and child as central to the development of the infant’s internal world. He described “primary maternal preoccupation,” a phase in which the mother is extremely attuned to the needs and wants of the child (Winnicott, 1956). This emotional parenting state is “almost an illness,” which allows the mother to reciprocate to her infant’s needs (Winnicott, 1960, 1965). The manifestation of “primary maternal preoccupation” can be thought of as a physical, emotional, and *adaptive* process that allows a mother to both identify with her infant while also allowing the infant to develop his/her own self. This extreme amount of focus of the mother on the baby, is the first step of her evolving into a new mother, or a mother to her new child.

Furthermore, John Bowlby was the first to formulate attachment as the quality of relationship between mother and child, which begins during infancy and continues throughout life (Bowlby, 1969, 1972, 1973, 1980, 1988). Decades of research have corroborated the central tenets of his attachment theory showing that emotionally sensitive and responsive mothers are more likely to have infants with secure attachment (Siegel, 1999; Insel and Young, 2001; Sroufe, 2005; Shah et al., 2010). These attachment patterns, which begin as early as infancy, sets a basis for the way adults interact, choose romantic partners, fall in love, and perhaps most importantly, parent their own children. An attuned and predictable attachment experience between the mother and child is therefore fundamental to the development of social, emotional, and psychological health.

Attachment theory is also important for understanding how individuals develop patterns of self-protective strategies, especially when faced with threats. The DMM model of Attachment and Adaptation (Crittenden, 1992, 1995, 2000, 2006; Crittenden and Landini, 2011) is a theory of attachment that is based on the work of Ainsworth and Bowlby but places a large emphasis on the role of danger, including separation and loss, in the development of specific behavior patterns. This theory of attachment posits that ongoing change is constantly interacting with experience to influence later development, and that any kind of threat or danger is powerful in organizing behavior (Crittenden, 2008). The DMM thus views attachment as a self-protective strategy (more so than security) which promotes the survival of a species (Crittenden and Landini, 2011), taking into account the adaptive skills of individuals in dangerous contexts. Thus, an attuned and predictable attachment experience between the mother and child is fundamental to the child’s future development of social, emotional, and psychological health when in a safe context.

### Neuroscience Perspectives

In addition to the psychological changes from an attachment perspective, motherhood is also characterized by neurobiological and hormonal changes that help establish maternal caregiving behaviors (Kim et al., 2010; Strathearn, 2011; Swain et al., 2014). Neuroimaging studies have demonstrated that the maternal brain undergoes significant structural changes during pregnancy and postpartum. Pregnant first-time mothers were observed to show significant decreases in their gray matter volume compared to their pre-pregnancy volumes (Hoekzema et al., 2017). This structural change was observed specifically in brain areas implicated in social cognition and theory of mind, such as the inferior frontal gyrus and precuneus, and possibly reflecting a specialized pruning which may occur in the expectant mother’s brain during pregnancy (Schurz et al., 2014; Hoekzema et al., 2017). Kim et al. (2010) further reported structural changes in the maternal brain during the initial postpartum months. Increases were seen in gray matter volumes of brain areas associated with the expression of maternal behaviors, including the bilateral hypothalamus, amygdala, substantia nigra, and globus pallidus. Increased gray matter volumes seen in these brain areas were associated with positive perception of infants by their mothers. Thus, structural changes in the maternal brain pre- and post-pregnancy are critical for the mother-infant bond.

A growing body of research has addressed attachment-related brain networks that govern maternal response to infant cues. Our prior work (Strathearn et al., 2009) has demonstrated that first-time mothers with secure patterns of attachment show enhanced activation of the key dopamine-associated reward-processing brain regions when viewing happy faces of their own infant. It was hypothesized that this may promote bond formation and attuned caregiving behaviors in these mothers. While mothers with secure attachment demonstrated similar dopaminergic reward responses when seeing their infant’s sad faces, mothers with insecure/dismissing attachment demonstrated an enhanced activation in the anterior insular region which is often associated with feelings of pain or disgust (Montague and Lohrenz, 2007). This underscores the importance of attending to the mothers’ own attachment history in understanding mother-infant outcomes, as this may influence her neural and behavioral responses to her infant, importantly shaping infant outcomes. In the section that follows, we examine individual differences in the mother’s attachment patterns and trauma history.

## Maternal Trauma and Its Intergenerational Transmission

### Attachment Perspectives

As discussed earlier, pregnancy and early postpartum are crucial periods during which a mother renegotiates her own identity as well as representations of herself and others (Slade et al., 2009) in order to prepare for a unique relationship with her new child. This transition that takes place during pregnancy and the postpartum period is particularly important for parents who have histories of psychological trauma which negatively influences their present experience. A seminal paper by Fraiberg et al. (1975) described this influence of memories or traumas in the past as “ghosts in the nursery.” These “ghosts” refer to emotionally painful memories experienced by the parent, which linger and impede their ability to sensitively respond to their own child, with the process being likely perpetuated across generations. Fraiberg’s work, while evocative, mirrored the idea that a parent’s inner world influences the child, and lays the foundation for understanding maternal mental health and infant outcome.

Exposure to trauma may lead to several possible outcomes. One is that an individual may garner new information from the situation and integrate the information into the present situation, resulting in adaptation and a newfound understanding that eventually helps the individual to avoid future danger. These individuals would be considered “resolved” with regard to the trauma or loss. In another scenario, an individual experiencing trauma or loss may either retain too much information about the traumatic event or dismiss the importance of it, in ways that are maladaptive to future processing of information (Crittenden and Landini, 2011). These individuals will be classified as having unresolved trauma, which refers to an individual’s maladaptive psychological response to a dangerous event that continues to adversely affect the individual’s strategic functioning (Crittenden and Landini, 2011). For example, mothers with what is termed a “preoccupied unresolved trauma” may maintain a focus on bringing their past trauma to the present and exaggerating emotions or affect, whereas mothers with a “denied or blocked unresolved trauma” may rely on omitting details or feelings associated with the past trauma (Crittenden and Landini, 2011). While both types of traumas have associated behaviors which allow the mother to protect against overwhelming feelings, such patterns of behavior may become maladaptive when directly applied to mother-infant relationships. Perhaps a mother with preoccupied unresolved trauma may be hyper-vigilant in response to her infant’s distress, while a mother with a denied unresolved trauma may under-respond to her infant’s distress.

The literature discussed above parallels the larger trauma literature both within and outside of the attachment framework. Specifically, two behavioral subtypes of trauma have been identified, hyperarousal and dissociation (Perry et al., 1995; Schore, 1997). While hyperarousal is defined by a heightened emotional and physiological response to trauma, dissociation represents a disconnection from or shutting down of the traumatic memory (American Psychiatric Association, 2013, DSM-V). Frewen and Lanius (2006), described the hyperarousal state as “reliving traumatic events in the form of flashbacks and experience-associated psychophysiological hyperarousal,” and Lanius et al. (2005) described the dissociative state as “an escape from the overwhelming emotions associated with the traumatic memory.” As a construct that has some overlap with unresolved trauma, PTSD has been shown to increase the risk for later psychosocial problems in affected individuals (Cicchetti and Toth, 1999; Easterbrooks et al., 2000), while also negatively affecting their caregiving of their own children.

Past research has identified the role of unresolved trauma in a range of psychiatric disorders, including borderline personality disorder (BPD) and substance use disorders (SUD). Prior work has shown that compared to normative mothers, mothers with BPD report higher instances of childhood abuse and neglect, display a higher prevalence of unresolved trauma, and demonstrate diminished reflective capacity (Fonagy et al., 1996; Barone, 2003), while also displaying exaggerated affect as well as signs of dissociation (Sieswerda et al., 2007; Scalabrini et al., 2017). Similarly, SUD has been strongly linked to the presence of adverse childhood experiences, high prevalence of unresolved trauma, and impaired parenting behavior (Rodning et al., 1991; Dube et al., 2003). From the perspective of attachment and trauma, key clinical features of BPD and SUD may be understood in light of the aforementioned subtypes of trauma (i.e., hyperarousal vs. dissociation and preoccupied vs. denied unresolved trauma) that may underlie these clinical presentations (Jacobsen et al., 2001; Watson et al., 2006; Sieswerda et al., 2007; Najavits and Walsh, 2012; Scalabrini et al., 2017). The presence of the unresolved trauma, in turn, impairs parental attunement and behavior (Rodning et al., 1991; Dube et al., 2003) when these individuals transition to motherhood.

### Neuroscience Perspectives

The presence of unresolved trauma not only visibly affects the mother’s attachment behaviors and emotional attunement but also the more invisible neurobiological responses to her infant’s cues. One such brain structure that is involved in the processing of trauma is the amygdala, which shows both structural (Rauch et al., 2000) and functional changes in the aftermath of trauma (Rauch et al., 2000; Protopopescu et al., 2005; Williams et al., 2006a,b). Specifically, neuroimaging studies examining the two subtypes of trauma have shown that distinct patterns of neural activation are associated with the two subtypes (Lanius et al., 2006; Hopper et al., 2007). Trauma patients with hyperarousal symptoms show an increased activation in the amygdala while showing a decreased activation in brain areas that are associated with emotion regulation (Lanius et al., 2006). In contrast, patients who exhibit dissociative symptoms show an increased activation in brain regions associated with emotion regulation while showing decreased activation in the amygdala (Lanius et al., 2006).

Previously we have explored the impact of unresolved trauma on amygdala response when first-time mothers viewed sad images of their infants (Kim et al., 2014). In this study, we found that mothers who were classified as having unresolved trauma displayed reduced activation in the amygdala, in response to seeing their infants in distress. This blunted amygdala response was seen only when these mothers viewed their *own* infant’s distressed face, and not that of unknown infants. This may reflect traumatized mothers’ disengagement from their infants’ distress, which could contribute to the intergenerational transmission of trauma. While our findings appear to be generally in line with the dissociative subtype of trauma, it is important to note that other groups have reported increased amygdala response to infant distress cues in mothers displaying disrupted attachment, possibly reflecting their hyperarousal (Riem et al., 2012).

Neurobiological studies on mothers with psychiatric disorders may further shed light on the ways in which the mother’s mental health including her unresolved trauma influences her brain responses to infant cues. Although individuals with BPD have been observed to demonstrate heightened activation of key emotion processing regions of the brain when presented with generic emotional cues (Lynch et al., 2006; Buchheim et al., 2008; Koenigsberg et al., 2009), no study has directly examined maternal brain responses to infant’s emotional cues in these individuals. However, there is a growing literature on the role of SUD in maternal brain response to infant cues. In a recent study, we examined brain responses of mothers with SUD and found that viewing happy images of their own infants resulted in a striking pattern of decreased activation in brain regions associated with reward and maternal caregiving, including the hypothalamus, ventral striatum, and medial prefrontal cortex (Kim et al., 2017). This reduced activation in key reward regions of SUD mother’s brains is particularly striking when considering that such blunted responsiveness was observed in reaction to the smiling faces of the mother’s own infant — likely the most rewarding cue that a mother will receive from her infant. While this may reflect a broader compromised caregiving that has long been reported for mothers with a history of substance use, it is important to note that 98% of the SUD mothers in this sample had a history of unresolved trauma. This raises the possibility that blunted maternal brain response to infant affect cues that we observed in SUD mothers may at least partly reflect the presence of unresolved trauma in these mothers (in line with the blunted responsiveness to salient affective cues that has been linked to a subtype of unresolved trauma).

In summary, studies from both attachment and neuroscience perspectives have demonstrated how unresolved trauma can interfere with a mother’s ability to sensitively respond to her infant. Furthermore, studies identifying the two subtypes of trauma, hyperarousal and dissociation, have provided critical insight by outlining specific ways in which unresolved trauma may manifest in mothers and disrupt her caregiving for her children.

## Protecting Against Intergenerational Transmission of Trauma

### Mentalization, Reflective Functioning and Attachment Reorganization

When Fraiberg et al. (1975) conceptualized a mother’s traumatic experience of the past and the “ghosts in the nursery,” they initially suggested that those mothers who processed and worked through their past traumas were less likely to transmit their trauma to the next generation and more likely to protect their future relationships with their child. Empirical research has since identified maternal factors that have been shown to protect against the intergenerational transmission of trauma, primarily centering around mentalization and reflective functioning (Fonagy et al., 1991a,b; Fonagy, 2002). Mentalization refers to one’s psychological ability to understand and interpret one’s own and others’ behavior as an expression of mental states such as feelings, thoughts, fantasies, beliefs and desires (Fonagy et al., 1991a; Fonagy, 2002). A closely related term reflective functioning is operationalized as a socio-cognitive capacity to mentalize in attachment-related contexts (Fonagy et al., 1991b; Allen et al., 2008; Ensink et al., 2014). Seminal work by Fonagy and colleagues showed that mothers who experienced substantial past trauma, yet were still highly reflective, were still able to have securely attached children despite their trauma (Fonagy et al., 1991a, 1995), while mothers with diminished reflective capacities went on to have insecurely attached children. This provided the first step in establishing a protective factor hypothesis for reflective functioning-suggesting that reflective functioning may halt the intergenerational cycle in situations where insecure attachment would have repeated across generations.

As described previously, attachment reorganization is another construct that has been identified as a potential protective factor against the intergenerational transmission of trauma (Crittenden, 1995, 1997). The construct of attachment reorganization is derived from the AAI (Crittenden, 1995, 1997) and is identified through a modified DMM-AAI coding scheme. The AAIs of reorganizing individuals reflect their capacity to alter their process of thinking from their self-protective attachment strategy, toward a more adaptive and reflective stance (Crittenden, 2017). While it is not necessary for an individual to experience danger or trauma before undergoing reorganization, the experience and resolution of danger can at times result in an alteration in mental processing and behavior, leading to a change in attachment strategy (Crittenden, 2008). To demonstrate reorganizing patterns, individuals need to be first aware of their discrepant patterns of thinking and altered behavior when they experience danger or trauma. Subsequently they need to work on applying change toward a healthy outlook related to the trauma. However, the individual may not be able to enact this change completely. Therefore, reorganization is a fluid process but one which holds promise to correct maladaptive patterns of thinking (Crittenden and Landini, 2011). During the AAI, reorganizing speakers make reflective and evaluative statements indicative of mental balance, incorporate new information to achieve a new understanding of situations, consider alternate perspectives, and achieve a cooperative relationship with the interviewer to find meaning in their history. Yet, there are slips into the dominant pattern of insecure attachment, and some level of unresolved incoherence noted in the discourse, as the reorganizing speaker attempts to reach an integrative conclusion about their situation.

### Earned Security and Attachment Reorganization

Another closely related and overlapping construct is that of earned security. Some theorists have used the term “earned secure” to refer to individuals who have experienced suboptimal parenting or adverse life events, but are able to overcome the effects of these experiences, demonstrate balanced integration, and attain secure attachment later in life (Pearson et al., 1994; Roisman et al., 2002). Earned secure individuals are thought to have interrupted the intergenerational cycle by demonstrating emotional resilience (Fearon et al., 2010). In comparison to earned security, reorganization encompasses the *process* by which those with insecure attachment or unresolved trauma are actively changing their understanding of past and present experiences in the direction of greater balance, resolution, and attachment security (Crittenden and Landini, 2011). Although, not fully secure or balanced in attachment terms, reorganizing individuals demonstrate the transient capacity to re-evaluate their history with a self-evaluative and reflective perspective (Landa and Duschinsky, 2013), which may have positive effects on the security of their progeny. Therefore, attachment reorganization captures a transient process, rather than a socio-cognitive capacity (e.g., mentalizing or reflective functioning) or a final stage of security (e.g., earned security). Dangerous circumstances may not allow the opportunity for self-awareness, nor may it be adaptive for survival. For example, leaving out important details of a trauma or keeping a trauma present at all times may function as self-protective and useful for individuals who find themselves in circumstances which threaten their survival. Therefore, attachment reorganizing is a novel concept in the attachment literature and potentially a more useful concept for individuals who exist in highly dangerous environments and lack the ability to accurately self-reflect.

### Clinical Examples of Reorganization

Our previous work (Iyengar et al., 2014) corroborated the intergenerational transmission of insecure attachment previously established in the literature, where mothers with unresolved trauma had insecure attachment themselves and were more likely to have infants with insecure attachment. Uniquely, 100% of the mothers with unresolved trauma who were reorganizing toward secure attachment had infants with secure attachment (although our sample size was small and this group encompassed only 4 infants). This illustrated the potential for change in attachment strategies over time and across generations, as well as the possibility of “attachment reorganization” (or reorganizing) as a protective factor. Our findings suggest that, for a mother who has an insecure pattern of attachment based on her childhood experiences as well as unresolved trauma, the fact that she is reorganizing toward secure attachment is just as advantageous to the intergenerational outcome as her being securely attached. Furthermore our work suggests that a mother with unresolved trauma does not necessarily need to reach the stage of earned security to halt the intergenerational transmission of trauma. The fact that she is in the process of reorganizing toward secure attachment may be enough to mitigate the disrupted relationship between the mother and her infant.

As previously stated, the DMM coding of the AAI regards the presence of unresolved trauma as a discrepancy in how they process information and events, noted in their discourse when asked about specific events and memories (see Iyengar et al., 2014 for more details on discourse and classification on unresolved trauma and reorganizing). The following examples are from the AAIs of two mothers with SUD, Jenny and Blair (names have been changed), who both display insecure attachment and the presence of unresolved traumas. Jenny is reorganizing toward secure attachment, while Blair is not.

#### Examples of Reorganizing and Non-reorganizing in Discourse

When asked to reflect on their experiences in the final integrative section of the AAI, we can see how Jenny, a reorganizing speaker, attempts to understand her father’s behavior, while Blair finds it difficult to accurately summarize her feelings and thoughts regarding her brother’s death.

     *Interviewer: Why do you think your father acted as he did during your childhood?*

     *Jenny: Because my dad was raised by the same kind of father. His father was an alcoholic, he was abusive… he’s never talked to me but I can imagine, that’s how it turned him into what he is. So that’s why I can forgive him, because I know that he didn’t just wake up one day and say, I want to beat my wife and make my kid’s lives miserable. He didn’t, you know, mentally he had been hurt emotionally.*

     *Interviewer: Are there things you wish to do with your child that are different to what your parents did?*

     *Jenny: Oh yeah, almost everything. You know, not the loving and affectionate part, cause I’m like that with my kids, I kiss them all, hug them, I love them, but making them feel bad about what they do, or not praising them for good things, um, hitting them, stuff like that. Everything that I didn’t, you know, that hurt me as a child, I don’t do with my kids because I remember that feeling.*

When reflecting on her past, Jenny includes an awareness that appearances are not always synonymous with reality. We can note a change in perspective, including past misunderstandings and reasonable current understanding *(you know, mentally he had been hurt emotionally*). Jenny also takes a reflective stance with evidence of taking in and using new information to arrive at new understandings (*Because my dad was raised by the same kind of father; he didn’t just wake up one day and say, I want to beat my wife and make my kids’ lives miserable).* She also demonstrates active efforts to tie past and present together in a psychologically sound manner (*Everything that I didn’t, you know, that hurt me as a child, I don’t do with my kids because I remember that feeling).*

However, when asked integrative questions that require a reflection on her childhood experiences, we can see how Blair struggles to reflect on her past and future, as seen in this example:

     *Interviewer: And now that you’re an adult, are there things that you want to do with your children that are different than what your parents did with you?*

     *Blair: Yes. Cause I don’t want them to end up like me, you know. I don’t want them to have to go through rehab twice. You know, it’s a struggle with life. And I think as much as I’ve been through, like just from the drug life, that what don’t kill you makes you stronger. I mean nothing bad, I’ve never had anything tragic, like I’ve never been abused, none of that, but just like struggling, because you spend all your money on drugs and your parents aren’t there for you no more, and just like having that feeling that hurt.*

Blair’s lack of reflection shows that she did not combine old and new ways of thinking about her traumatic experiences to derive accurate thoughts about the future. Rather, she demonstrated the use of optimistic platitudes (*what doesn’t kill you makes you stronger*), and while she demonstrates a hopeful wish for her children (*I don’t want them to end up like me)* there was no conclusive integration that indicated that connections were being made to elicit change. Blair’s discourse is in contrast to the several reorganizing aspects of Jenny’s discourse.

Taken together, both Jenny and Blair had unresolved trauma and/or loss, as well as insecure attachment, while only Jenny was reorganizing toward secure attachment. It is important, then, to note that a mother with a pattern of thinking like Jenny’s, demonstrates the potential to re-evaluate the past and present and come to new conclusions that may benefit the relationship with her child. In comparison, a mother with a pattern of thinking like Blair’s, may not be able to accurately reflect on the needs of her children and may misinterpret or deny their distress, just as she did her own, and may require more support or encouragement to process her past experiences and reflect on the present to embody change.

## Conclusion

The transition to motherhood is characterized by dramatic changes which prepare the new mother for the optimal rearing of her young. It is a critical period that is both adaptive and conducive to establishing a secure mother-infant relationship. Despite being rewarding, motherhood comes with its own set of challenges, more so for mothers with unresolved trauma. In this review, we have introduced and expanded on the concept of attachment reorganization and evaluated its potential as a protective factor in mothers with unresolved trauma. We have also discussed how the novel concept of attachment reorganization differs from other overlapping concepts of mentalization, reflective functioning, and earned security. The concept of reorganization encapsulates the process of change from insecure attachment to secure attachment, and from unresolved trauma to resolved trauma. This parallels the process of reflection, re-evaluation, and change that often takes place in psychological treatments, but is unique in that it captures the process unfolding specifically in relation to one’s past attachment experiences and has promise in positively shaping intergenerational attachment outcomes.

Although novel, promising and indicating potential for clinical implications, the construct of reorganization is relatively new and has not been subject to much empirical examination. For the construct to be widely used in empirical research, additional data is needed on its psychometric properties, including its construct validity and reliability. Future research should examine this novel construct as a critical protective factor that may halt the intergenerational transmission of insecure attachment and trauma. Examining reorganizing in clinical sample of mothers (e.g., mothers with SUD or BPD) would further help researchers and clinicians understand what difference reorganizing toward secure attachment makes in mothers who have psychiatric symptoms involving unresolved trauma.

It is especially valuable to examine attachment reorganization during pregnancy or in the early postpartum, as it captures the adaptive capability of the human brain and behavior in a measurable attachment-related construct. Perhaps, reorganizing in an attachment context, corroborates what we already know about the brain’s ability to form new connections between neurons and change throughout life, with the brain never being fully organized, yet always in the dynamic and adaptive process of change.

## Author Contributions

UI, PR, PF, LS, and SK conceived and designed the manuscript. UI and PR wrote the manuscript with input from all authors. All authors added critical insight and intellectual contribution to the overall work, and SK supervised the procedure.

## Conflict of Interest Statement

The authors declare that the research was conducted in the absence of any commercial or financial relationships that could be construed as a potential conflict of interest.
